# Refinement of a workflow for human relevance assessment of adverse outcome pathways and associated new approach methodologies

**DOI:** 10.3389/ftox.2025.1616817

**Published:** 2025-08-04

**Authors:** Annick D. van den Brand, Julia J. Meerman, Christina H. J. Veltman, Mirjam Luijten

**Affiliations:** Centre for Health Protection, National Institute for Public Health and the Environment (RIVM), Bilthoven, Netherlands

**Keywords:** human health risk assessment, chemicals, toxicity pathways, adverse outcome pathways, new approach methodologies, next-generation risk assessment, human relevance assessment

## Abstract

In chemical risk assessment the human relevance of adverse health effects observed in experimental animal studies and the underlying toxicological mechanisms, i.e., adverse outcome pathways is often assumed, unless evidence suggests otherwise. Yet, detailed systematic guidance as to how human relevance of perturbed AOPs should be assessed and which data or information should be considered is lacking. Building on previous work we present a refined workflow for human relevance assessment of AOPs and associated new approach methodologies The updated workflow structurally defines the required information for assessing the human relevance of the AOP by means of biological and empirical considerations. Furthermore, the modified workflow better guides assessment of the relevance of NAMs. This is of importance for the use of NAM data in human health risk assessment. In addition, we suggest an approach for weight of evidence assessment by integrating the different lines of evidence. The refined workflow is now accompanied by developed guidance and templates as well as an expanded toolbox, i.e., a list of information sources, to further facilitate application of the workflow. Finally, remaining issues and challenges are discussed. This work is a next step towards to the ultimate goal of a harmonized, structured and transparent approach for human relevance assessment of AOPs and associated NAMs.

## 1 Introduction

With the ambition to reduce, refine and replace experiments relying on laboratory animals for the safety assessment of chemicals and pharmaceuticals, novel approaches, methods and techniques are increasingly being developed. These so-called new approach methodologies (NAMs) are by many considered the future of human relevant chemical hazard and risk assessment, coined as next-generation risk assessment. It is to be expected that in the coming years data obtained from NAMs will increasingly be used in human health risk assessment of chemicals. In the context of this work, we define NAMs as any alternative (non-)test method or methodology that can be used to provide information on chemical hazard and risk assessment in line with the Interagency Coordinating Committee on the Validation of Alternative Models (ICCVAM, 2018).

It is pivotal for human health risk assessment that the relevance of data based on *in vivo*, *in vitro* or *in silico* toxicity studies is assessed. This applies to postulated toxicological pathways (in this work further referred to as adverse outcome pathways (AOPs)) that describe the underlying mechanism for a given adverse health effect as well as to NAMs that are associated with the different elements of such pathways. In order to structure and support human relevance assessment, the mode of action (MOA)/human relevance framework was developed by initiatives of the International Programme on Chemical Safety (IPCS) of the World Health Organization (WHO) (WHO/IPCS, 2007). According to the initial versions of that framework, human relevance assessment considers whether evidence assembled for a postulated MOA carries sufficient weight to consider the MOA relevant for humans. That framework considers the similarities regarding both the qualitative aspects and quantitative aspects of biological processes in human and animals. In 2014, the MOA/human relevance framework was updated to extend its utility to emerging areas in toxicity testing, i.e., the use of NAMs (Meek et al., 2014a; Meek et al., 2014b).

The main questions that need to be addressed to assess human relevance using the WHO/IPCS MOA/human relevance framework are:

• Is the weight of evidence sufficient to establish a MOA in animals?
• Can human relevance of the MOA be reasonably excluded on the basis of fundamental, qualitative differences in key events between experimental animals and humans?
• Can human relevance of the MOA be reasonably excluded on the basis of quantitative differences in either kinetic or dynamic factors between experimental animals and humans (WHO/IPCS, 2007)?

To exemplify how the WHO/IPCS MOA/human relevance framework could be applied, several case studies have been reported. These case studies cover different health effects and different classes of chemicals (e.g., Meek et al., 2003; Kavlock and Cummings, 2005; Zoeller and Crofton, 2005; Cohen et al., 2006). As to how human relevance of a MOA can reasonably be excluded, the framework also provides examples of types of information that can be useful to include in the assessment, as illustrated by various case studies (Meek et al., 2003; Rasoulpour et al., 2014; Strupp et al., 2016; Frank and Meek, 2024).

Despite the validity of the questions and the approach used in the MOA/human relevance framework, its application can be improved by systematic guidance as to how to answer these questions and which aspects to consider. Additionally, a specific and dedicated human relevance assessment is currently not required in the development of AOPs according to the OECD Guidance (OECD, 2018). Furthermore, besides the human relevance of the AOP itself, another important issue is the relevance of NAMs that are associated with the different elements, such as key events (KEs) or key event relationships (KERs), of the respective AOP. According to OECD Guidance Document 34, “(…) the relevance of a test method describes the relationship between the test and the effect in the target species and whether the test method is meaningful and useful for a defined purpose, with the limitations identified” (OECD, 2005). It is too simplistic to assume that NAMs using cells of human origin are automatically relevant for human health risk assessment. This relevance depends on the specific biological context and the associated type of information that the NAM can provide. For example, aspects like required metabolic competence, p53 functionality or proliferation rate may be of importance for the applicability domain of one NAM, but not for another. A better insight into the relevance of NAMs in the context of a given AOP will greatly advance the transition of traditional animal-based testing towards NAM-based testing for regulatory purposes. Therefore, Veltman et al. (2025) developed a workflow for the assessment of human relevance of an established AOP as well as its associated NAMs, based on the WHO/IPCS MOA/human relevance framework. Veltman et al. (2025) provide an initial implementation of the workflow to assess human relevance, as illustrated with a case study on “Disruption of retinoic acid metabolism leading to developmental craniofacial defects” (Menegola et al., 2021). Moreover, a toolbox was created providing an overview of various information sources that can be considered when assessing specific aspects of human relevance, for example, the human protein atlas,^1^ expression atlas^2^ and ENCODE project.^3^ Despite the fact that we focus in the context of this work on NAMs, the use of data from traditional toxicity tests in experimental animals should, where relevant, not be excluded in a human relevance assessment.

Although the workflow as described by Veltman et al. (2025) provides a clear example of how human relevance could be assessed using the workflow, a structured guidance on the workflow and templates for the application of the workflow are lacking. Here, we evaluate and refine the human relevance workflow as described in Veltman et al. (2025) by practically applying it, with a focus on the first (out of three) question. Additionally, we developed guiding templates. Such guidance, including an approach for weight of evidence evaluation and a suggestion on how to merge various lines of evidence is imperative for a wider application of the workflow. Below, we address the main modifications made to the workflow by Veltman et al. (2025), describing the developed guidance to help assess whether an AOP relevant to humans.

## 2 Methods

**FIGURE 1:**
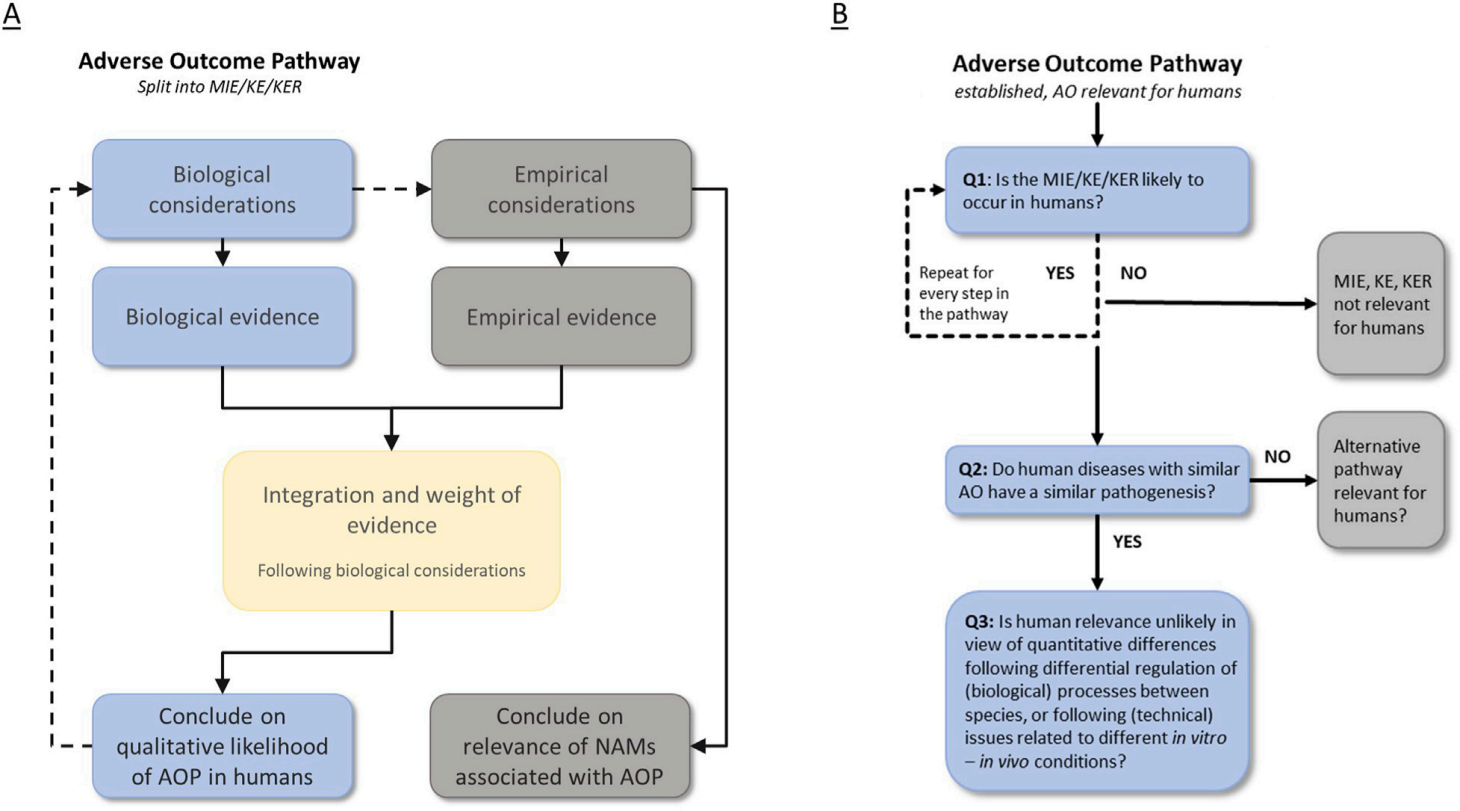
Schematic overview of the workflow for the assessment of human relevance of an AOP and its associated NAMs. The starting point of the workflow for human relevance assessment is an established AOP. Panel **(A)** overview of the human relevance assessment and information types that result in: 1) a conclusion on the qualitative likelihood of an AOP in humans and 2) a conclusion on the relevance of NAMs associated with the respective AOP. Panel **(B)** overview of the modified workflow with its specific questions. AO: adverse outcome; KE: key event; KER: key event relationship; MIE: molecular initiating event.

We applied the human relevance workflow as described by Veltman et al. (2025), where we focused on the first question; whether it is likely that an AOP (or elements thereof) can (qualitatively) occur in humans. The main goal of this work was to test the applicability of the workflow and, based on the lessons learned, refine the workflow, improve the toolbox for sources of information and develop guidance and templates. The starting point of the workflow is an established AOP, with an endpoint that is considered of relevance for human health risk assessment. By applying the workflow to an AOP, two types of data are assessed–biological evidence and empirical evidence – that are greatly dependent on each other. The end result of applying the workflow is 1) a conclusion on the qualitative likelihood of an AOP in humans; and 2) a conclusion on the relevance of identified NAMs associated with the individual elements of the AOP that can be used to provide data relevant for human health risk assessment (Figure 1A).

In this study, by applying the workflow using two established AOPs, we identified various limitations of the workflow and made modifications accordingly. We applied elements from AOP#3 “Inhibition of the mitochondrial complex I of nigro-striatal neurons leads to parkinsonian motor deficits” (AOPWiki, 2023b) and AOP#220 “Cytochrome P4502E1 activation leading to liver cancer” (AOPWiki, 2023a).

Below, an updated version of the workflow (Figure 1B) and toolbox is described. Additionally, we developed guidance and templates accompanying the workflow to aid the human relevance assessment of an AOP and associated NAMs Supplementary File 1.

## 3 Results

The starting point of the workflow for human relevance assessment is an established AOP (Figure 1). We consider an AOP established when the overall weight of evidence for the AOP is at least moderate, i.e., sufficient (empirical) evidence is available to support the biological plausibility of the KERs and the essentiality of the KEs based on the modified Bradford Hill criteria (OECD, 2017). We thereby skip the first question of the WHO/IPCS MOA/human relevance framework, which addresses the toxicological pathway itself (MOA), to solely focus on the human relevance aspect. In the workflow, the first step addresses the question whether the individual elements of the AOP under consideration, i.e., the molecular initiating event (MIE), KEs and KERs, are qualitatively likely to occur in humans. Such an evaluation should be made for all elements in the AOP. In the original workflow, an additional branch was incorporated for scenarios where there was insufficient data. A sub-question related to evolutionary conservation then had to be answered. Considering this information is also relevant when other information is available and to maximize the applicability of the workflow, this sub-question is now incorporated as a consideration in the first question’s guidance (see below). The revised figure of the workflow is depicted in Figure 1B.

### 3.1 Considerations

When evaluating the different elements in an AOP with regard to human relevance, both biological and empirical aspects should be considered. These considerations serve the identification, collection and organization of the data that is needed to answer the question on human relevance of the AOP. Biological considerations provide information with respect to the underlying biology of the chain of events and their relationships. Empirical considerations provide information with respect to the empirical evidence that is provided to describe the chain of events in the AOP, obtained from, e.g., NAMs. These two lines of evidence are dependent on each other. The underlying biology is an important aspect to evaluate when assessing whether NAMs provide evidence that can be considered relevant for the respective AOP. Data from NAMs, when indeed considered to provide relevant evidence, can be used to support the biological evidence that conclude on the human relevance of an AOP. Below we further specify these considerations.

#### 3.1.1 Biological considerations and toolbox

To answer the question whether the elements of an AOP (which can be a KE and/or KER) are likely to occur in humans, information on various biological aspects of human relevance needs to be systematically gathered. We now refer to these aspects as biological considerations and regard this as the first part of the workflow (Box 1).

**Box 1:** Original and modified biological considerations.

Original	Modified
1. Expression/occurrence in target organ	1. Expression/occurrence in target organ
2. Expression/occurrence specific to a sensitive time-window	2. Expression/occurrence specific to a sensitive time-window(s); and/or specific to life-stage
3. Expression/occurrence specific to gender	3. Expression/occurrence specific to gender/sex
4. Expression of isoforms	4. Relevant case reports, pharmacovigilance (clinical data)
5. Polymorphisms that may affect expression	5. Evolutionary conservation
6. Enzymes/metabolism that may affect KE/KER	
7. Relevant case reports, pharmacovigilance	

Veltman et al. (2025) describe various biological aspects of human relevance, such as target organ, sensitive time window(s), or life stage(s), and possible differential expression between sexes. We now grouped and refined the various aspects to be taken into account. In contrast to the original workflow by Veltman et al., we removed the considerations addressing the expression of isoforms, polymorphisms and enzymes (metabolism), which may affect the element(s) of the AOP under assessment (Box 1). These aspects are certainly important when assessing human relevance of an AOP, yet deemed more important for assessing quantitative differences between subgroups of the human population, and not qualitative relevance of (elements of) an AOP. In the current refined workflow, the biological considerations that are required for human relevance assessment of the AOP are (a full description is provided in the Supplementary Material).

##### 3.1.2 Expression/occurrence in target organ(s)

This consideration requests information on the actual expression or the occurrence of the element in the human target organ. Is there evidence that supports or does not support the element of the AOP (which can be a KE and/or KER) under assessment occurring in humans?

##### 3.1.3 Expression/occurrence specific to a sensitive time-window(s) and/or specific to life-stage

This consideration requests information on the expression or the occurrence of the element in the appropriate time-window and/or life-stage. Is there evidence available that supports or does not support the element under assessment occurring at the relevant time-window and/or life-stage?

##### 3.1.4 Expression/occurrence specific to gender/sex

This consideration requests information on the possible (lack of, or impaired) expression or occurrence of the element in human males or females. Is there evidence available that supports or does not support the element under assessment in one or both of the sexes? This impacts the information that is applicable to the other considerations as well.

##### 3.1.5 Relevant case reports, pharmacovigilance (clinical data)

This consideration requests information on the event in relation to relevant human case reports, pharmacovigilance or other clinical data. Is there evidence available that supports or does not support the element in the AOP under study in humans? Note that this does not necessarily require information on the human occurrence of the AOP as a whole.

##### 3.1.6 Evolutionary conservation

This consideration requests information on the evolutionary conservation of the element. If the conservation of the element is very high, expression in humans can be assumed. Is there evidence available that supports or does not support the element under assessment in different (e.g., mammalian/vertebrate/…) species?

In the original workflow, Veltman et al. (2025) created a toolbox providing sources for information that may be used to collect data on various biological aspects of human relevance. This toolbox was now modified to better reflect the biological considerations, and additional information sources were added (Supplementary Table S1).

In order to use the gathered information on these biological aspects of human relevance in the assessment, the information needs to be checked for relevance and reliability. Information from general sources, such as widely recognized text books, may be considered established information and, therefore, reliable. However, data from, e.g., single clinical studies need to be of sufficient quality and considered relevant and reliable to be used. For such assessment, various tools are available for clinical and animal studies and epidemiological data, such as the OHAT Risk of Bias Rating Tool and the Critical Appraisal Tools [CATs – for assessing quality of randomised controlled trials (RCT CAT) and systematic reviews of intervention studies (SR CAT)] as described by EFSA (EFSA, 2015; OHAT, 2015). Criteria regarding the study’s conduct and the directionality of potential bias must be critically reviewed. This step is highly important for data to be included in the assessment, but evaluating these tools is out of the scope of the workflow.

### 3.2 Empirical considerations

Each NAM that can be used as source of information should be assessed for its power to support human relevance in relation to the evaluation of an AOP or element(s) thereof. To do so, we provide additional and modified empirical considerations, following the considerations for empirical evidence as mentioned in the original workflow by Veltman et al. (2025) (Box 2).

**Box 2:** Original and modified biological considerations.

Original	Modified
1. Species	1. Appropriateness
2. Representativity	2. Complexity
3. Complexity, cell status	3. Sensitivity and susceptibility
4. Directness of evidence	
5. Robustness	

These considerations reflect aspects that need to be taken into account when using empirical data from NAMs to assess human relevance of an AOP. The empirical considerations are provided to structure the information and systematically assess the relevance of each NAM or other (experimental) model in the context of the AOP under study. It is not meant to (dis)qualify NAMs in general; the purpose rather is to assess their relevance in the context of the AOP studied. In contrast to the original workflow by Veltman et al. (2025), we removed the considerations that addressed robustness and validity of the NAMs (Box 2). Robustness or reproducibility of the (non-)test method is a consideration that needs to be evaluated in general, yet does not affect its relevance evaluation in this context (OECD, 2005). In the assessment it should be described what is known on the established use and readiness level of the test method and, if applicable, the protocol or standard operating procedure of the test method should referred to. Also, validity of a test method is important, yet does not affect the relevance of a method in the context of an AOP *per se*. As goes for the robustness of the test method, we assume a basic level of validity for the selected NAM. It is assumed that the data can be reproduced within and between laboratories, and that the data are reliable and relevant. In other words, we do not *a priori* question the exposure scenario used, the method setup, etc.

The empirical considerations that are required to be included in the workflow for relevance assessment are as follows (a full description is provided in the Supplementary Material).

#### 3.2.1 Appropriateness

This consideration requests information on the appropriateness of the test method and measured response in relation to the element under assessment. The test method needs to sufficiently represent the element under assessment and capture the appropriate model response. A range of questions was drafted that need to be considered for this line of thought. For example, is the appropriate organ or life-stage reflected in the test method and is the response that is measured in the test method directly related to the associated element of the AOP and reflecting the human *in vivo* situation? It also considers the possibility of metabolic activation, e.g., expression of relevant CYP enzymes as compared to the *in vivo* situation.

#### 3.2.2 Complexity

This consideration requests information on the complexity of the test method and its appropriateness for the element under assessment. The test method needs to be fit-for-purpose, i.e., sufficiently complex to measure/provide evidence for the element that is under assessment. Two questions were drafted that need to be considered for this line of thought: a) is the complexity of the test method sufficient for the level of biological organization of the element(s) under assessment (because, activation of a receptor or enzymatic catabolism/metabolism do not necessarily need to be demonstrated in an organ on a chip); and b) is the physiological state of the test method sufficiently captured (because, some KEs do not necessarily need to be demonstrated in a 3D model using multiple cell types)?

#### 3.2.3 Sensitivity and susceptibility

This consideration requests information on the sensitivity and susceptibility of the experimental model and its appropriateness for the element under assessment with the aim to identify gaps of analogy. Differences in, e.g., isoforms, polymorphisms, and transporter expression may affect the evidence that the test method can provide regarding human relevance and the translation of the results to the human population. Two questions were drafted that need to be considered for this line of thought: a) is the element under study appropriately expressed in the experimental model; and b) is an increased or decreased response expected as a result of sex/isoform/polymorphism differences?

We acknowledge that it can be more straightforward to evaluate the NAMs and respective considerations for the elements in the anterior part of the AOP. Evidence for, e.g., the activation of a receptor can be acquired using a relatively “simple” cell culture model, whereas events occurring at an organ level are usually more complex to simulate.

The toolbox provided in the section on biological considerations can also be of use here to gain information on the required biological aspects of relevance for the experimental model.

The evidence that is obtained from the NAMs that are considered sufficiently relevant can be included in the human relevance assessment. This evidence can be used to support the information related to the biological considerations for the respective elements of the AOP. It should be noted that the reliability and quality of these individual studies are essential and need to be assessed by available tools, e.g., the SciRAP tool or the ToxRTool for *in vitro* toxicity data prior to accepting the data in the human relevance assessment (Roth et al., 2021; EFSA et al., 2017b; Schneider et al., 2009). Criteria on, e.g., the conduct and reporting of the study guide the assessment of the reliability and quality of the study’s data in a transparent manner resulting in a final “score” or assigned Klimisch score. Although evaluating these tools is out of the scope of this work, we consider such assessments a mandatory step when using toxicological data in the assessment of human relevance.

### 3.3 Weight of evidence

To reach an overall conclusion as to whether the elements in an AOP can occur in humans, two types of evidence should thus be pursued (Figure 1A); a) information with respect to the underlying biology of the chain of events (biological considerations), and b) the empirical evidence from NAMs (or other test methods) that can be used to measure the different events (empirical considerations). Based on these bodies of evidence, a final conclusion can be made as to the qualitative human relevance of the (elements in an) AOP and the associated NAMs, which then can be used to demonstrate the effect of chemicals on this pathway in a human health risk assessment.

This biological and empirical evidence, therefore, needs to be systematically investigated to reach a transparent conclusion on the qualitative likelihood of the elements in the AOP to occur in humans (Q1 in the human relevance workflow). We propose that such systematic investigation is performed using a weight of evidence approach (EFSA et al., 2017b; Escriva et al., 2021; Wiklund and Beronius, 2022; OSHA, 2016).

The first step in a weight of evidence assessment is the definition of an assessment question, which was here defined as Q1 in the human relevance workflow. To answer this question, the biological considerations as defined above can be regarded as individual lines of evidence. Identified information on the biological plausibility of human relevance on the various elements in the AOP can be substantiated with empirical information obtained from NAMs. Theoretically, all information that is available, biological or empirical, needs to be considered in a human relevance assessment. Yet, in practice, when there is a significant amount of evidence supporting the biological plausibility, empirical evidence will most likely not significantly alter the assessment of human relevance. In contrast, in a scenario where evidence on biological plausibility is limited or lacking, empirical evidence is a prerequisite.

#### 3.3.1 Integration of the lines of evidence

To answer the assessment question, theoretically, both the biological and empirical evidence need to be considered. The integration of this evidence is part of the weight of evidence approach and the considerations as defined above – the lines of evidence – help to organize and weigh the evidence. We propose to integrate the evidence within each line of evidence in a categorical manner. Therefore, we have suggested a tabulated format with principles for categorization to assess the collected data in a categorical manner (Supplementary Tables S3, S5). This can help the assessor in combining the available information for each line of evidence. Direction is given to conclude on an assessment on human relevance in the context of the particular line of evidence. In addition, factors like consistency of the evidence, and quality of the evidence need to be considered here.

To integrate the evidence from all the considerations, i.e., all lines of evidence, we suggest using a general ‘rule of thumb’, to be applied on all elements that are under assessment for human relevance. For each element in the AOP a narrative conclusion should be drawn based on the whole body of evidence, where the biological evidence is leading. When all elements in the AOP have been assessed, an overall conclusion on the relevance of the pathway can be drawn.

For a next version of this workflow we propose to refine this weight of evidence analysis by refining/rephrasing the tables that are proposed for the categorization of the lines of evidence, if required, and by discussing with a broad group of experts the ‘rules of thumb’ for the integration of the lines of evidence. More considerations for the biological lines of evidence can be added where desired. In addition, some considerations may be preferred to carry more weight in the assessment as compared to other considerations. This can also be included in the weight of evidence analysis. It should, however, be noted that an optimum balance needs to be pursued between a weight of evidence assessment that is complex enough to add value and still be practical to work with.

### 3.4 Guidance and templates

In addition to the modifications that were made to the considerations of the original workflow, we created a structured guidance and template through the modified workflow of Veltman et al. (2025) (see Supplementary Material). Such guidance aids in the adoption, the application and the general use of the workflow.

In the guidance developed, we describe the steps that one can take for a structured and transparent assessment on human relevance. We provide more information on the biological and empirical considerations and have created tabulated formats where the information (i.e., evidence) for human relevance can be summarized. Summarized examples of information collected for both the biological and the empirical considerations are presented in Box 3 and Box 4. The example provided in Box 3 focuses on the AOP “Inhibition of the mitochondrial complex I of nigro-striatal neurons leads to parkinsonian motor deficits” (AOPWiki, 2023b), while the example shown in Box 4 is related to the AOP “Cyp2E1 Activation Leading to Liver Cancer” (AOPWiki, 2023a).

BOX 3 **Example assessment biological considerations.**

**FIGURE 2:**
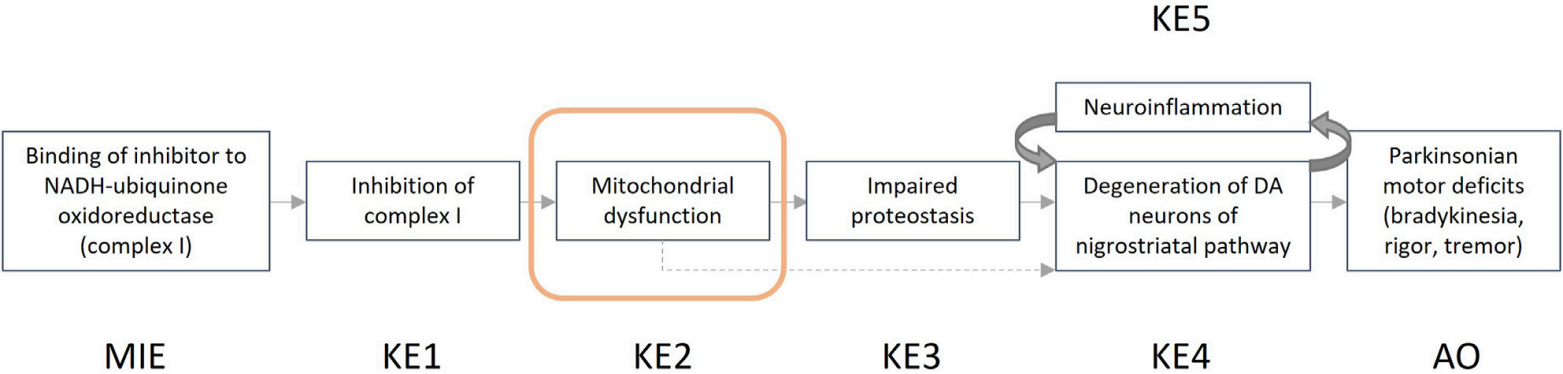
Graphical representation of the adverse outcome pathway “Inhibition of the mitochondrial complex I of nigro-striatal neurons leads to parkinsonian motor deficits.”

Here we evaluate the human relevance of the key event ‘mitochondrial dysfunction’, as part of AOP 3 and an AOP network (Meerman et al., 2023; Terron et al., 2018; AOPWiki, 2023b) ultimately leading to the adverse outcome Parkinsonian motor deficits (Figure 2). Dysfunction of the mitochondria in dopaminergic neurons of the substantia nigra is evaluated using the biological considerations.

**1. Target organ**: Substantia nigra dopaminergic neurons express mitochondria that can be inhibited *in vitro* by prototypic stressors as indicated by, e.g., reduced ATP levels and cellular respiration. It was shown that cellular ATP levels decreased in the SH-SY5Y and SK-N-SH cell lines (originating from human neuroblastoma) after exposure to Rotenone (Mader et al., 2012; Rekha and Inmozhi Sivakamasundari, 2018). A decrease in ATP levels is a direct indication of dysfunctional mitochondrial electron transport chain. Also, Trifluralin exposure resulted in impaired cellular respiration in human iPSC dopaminergic neurons (Paul et al., 2023), which indicates mitochondrial dysfunction. This evidence shows that mitochondrial disfunction can indeed occur in the target cells.

**2. Time window**: An age-associated decline in mitochondrial functioning has been reported in muscle and liver tissue (healthy elderly and young volunteers) (Petersen et al., 2003). Mitochondrial DNA (mtDNA) deletions have been reported in adult brain samples, but were not found in fetal brain samples (Cortopassi and Arnheim, 1990). Increasing mtDNA deletions may confer increased vulnerability of mitochondria due to ageing. The same mtDNA deletion was again reported to increase with age, and the highest frequency of deletions was found in three specific brain areas: the substantia nigra, caudate, and putamen (Soong et al., 1992). Similarly, the number of mtDNA deletions in substantia nigra was reported to be significantly different between old and young samples (Kraytsberg et al., 2006). In skeletal muscle tissue, mtDNA and mRNA abundance and ATP production declined with advancing age (Short et al., 2005). This leads to the conclusion that the KE can occur during all life stages, but is more likely to be adverse as age increases.

**3. Sex**: Mitochondrial function has been reported to differ between sexes, based on mitochondrial parameters from peripheral blood mononuclear cells and brain metabolites (Silaidos et al., 2018). There are other indications for sex differences in mitochondrial function and dysfunction, including mitochondrial quality control, mitophagy, and protective mechanisms to deal with excitotoxicity and oxidative stress (Demarest and McCarthy, 2015). However, many of the studies (if not all) cited used animal data. In PD patients, differentially expressed genes linked to mitochondrial activity were found to be increased in females. Authors suggest mechanisms underlying PD may involve mitochondria more in females as compared to males, although not exclusively (Lopez-Cerdan et al., 2022).

**4.** **Clinical data**: Several clinical trials have tried to target mitochondrial dysfunction to prevent PD progression, though with very limited success (Henrich et al., 2023). The lack of success may be in part due to poor drug delivery and failure to address the complexity of PD development by focusing solely on mitochondria (Prasuhn et al., 2020). Nonetheless, an impairment of mitochondrial function (as a result of reduced complex-I activity) was observed in substantia nigra in PD patients, but not all evidence hitherto is conclusive (Bose and Beal, 2016; Chaturvedi and Beal, 2008; Larsen et al., 2018). Genetic defects in the mitochondrial quality control genes (DJ1, PINK1, parkin) have been linked to familial cases of PD (Henrich et al., 2023). Further evidence of the critical role of mitochondria in PD is that parkinsonian symptoms (among other pathologies) manifest in patients with mutations in the mitochondrial polymerase gamma gene (Rahman and Copeland, 2019).

**5. Evolutionary conservation**: Mitochondria are present in virtually all mammalian cells considering their critical function in the cell’s energy generation (Bulthuis et al., 2019). The majority of energy of eukaryotic cells, in the form of ATP, is produced in the mitochondrial oxidative phosphorylation (OXPHOS) system (Zhang and Broughton, 2013). There are many similarities between the regulation of mitochondrial transcription DNA initiation, including the mitochondrial OXPHOS system, between yeast and human which highlight underlying evolutionary conserved elements and mechanisms (Basu et al., 2020). The synthesis of ATP can, *inter alia*, be inhibited by ATPase inhibitory factor 1 (IF1) thereby regulating mitochondrial respiration (Esparza-Molto et al., 2021). IF1 is highly conserved in mammals and yeast and the core metabolic and redox circuits that are modulated by IF1 are probably conserved across different cell types and brain regions (Cabezon et al., 2002; Esparza-Molto et al., 2021).

Taken together, the human relevance of the KE mitochondrial dysfunction in the context of this AOP is moderate to strong. For none of the considerations, weak evidence for human relevance was identified.

BOX 4 **Example assessment empirical considerations.**

**FIGURE 3:**
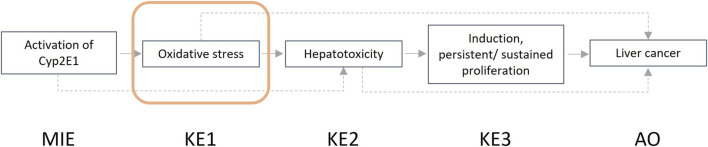
Graphical representation of the adverse outcome pathway “Cyp2E1 Activation Leading to Liver Cancer.”

Here we evaluate the relevance of two NAMs related to the key event ‘Oxidative stress’, as part of AOP 220 (Figure 3) and an AOP network (Veltman et al., 2023; AOPWiki, 2023a) ultimately leading to the adverse outcome liver cancer. Analysis of oxidative stress in zebrafish embryos (ZFE) and Upcyte human hepatocytes (UHH) are evaluated for their power to support human relevance assessment using the empirical considerations.

ZFE

**1. Appropriateness**: ZFE are embryos from zebrafish and allow for oxidative stress detection and quantification in a whole organism. The localization of oxidative stress, for example, in the liver, is possible, yet in this specific assay whole embryos are homogenized. Oxidative stress is analyzed at the end of the zebrafish’ developmental stage. In this assay, antioxidant capacity is analyzed by means of a glutathione reductase activity assay. This is an indirect measure for oxidative stress (Stevens et al., 2024).

**2. Complexity**: Suggested CYP2E1 homologues (relevant in this AOP as Cyp2E1 activation is the preceding KE) and antioxidants are present in zebrafish embryos, which are metabolically competent (Hartman et al., 2017). This assay assesses antioxidant capacity as an indirect measure of oxidative stress in a whole embryo (Stevens et al., 2024). Considering that embryos were homogenized, this assay cannot be used for localization purposes. Although this is a metabolic competent model, it is relatively complex to represent a cellular key event.

**3. Sensitivity/susceptibility**: A pool of male and female embryos were analyzed, although this assay does not specifically address sex-specific differences as the sex in zebrafish can be distinguished only after approximately 21 days post fertilization (von Hofsten and Olsson, 2005; Uchida et al., 2002). The exact genetic make-up of the AB (wildtype) strain is unknown. Affinity of CYP2E1 substrates in zebrafish can differ and enzyme activity is much lower in zebrafish compared to mammalian CYP2E1 (Hartman et al., 2017). Despite being from the same strain, the ZFE are more genetically diverse as compared to cell lines which originate from a single cell.

UHH

**1. Appropriateness**: UHH cells are derived from primary adult human liver cells. The cells can be cultured for a relative long period of time as compared to primary cells. The UHH were characterized by transcriptomics and their functionality was assed based on stable expression and activity of CYP enzymes (including the relevant CYP2E1 in this AOP) and antioxidants (Donato et al., 2022). Oxidative stress was appropriately analysed by means of reactive oxygen species (ROS) detection using CellROX staining.

**2. Complexity**: ROS are produced in mitochondria of, i.a., liver cells and can, therefore, appropriately be assessed in UHH. These cells are derived from primary human cells and are as such not a tumorigenic cell line but reflect a “normal” human liver cell. CYP2E1 is expressed and active in these cells and it was shown that ROS production can be increased by chemical exposure. This model is genetically more diverse and metabolically competent as compared to carcinogenic liver cancer cell lines. The application is limited to one organ, yet the relevant organ with respect to the KE under assessment.

**3.** **Sensitivity/susceptibility**: UHH from multiple donors can be mixed together or assessed individually to overcome or study age or sex-specific differences in the response, in contrast to, e.g., HepaRG cell line that is derived from one donor and does not reflect population differences (Donato et al., 2022). There is a decreased expression profile observed in CYP2E1 mRNA and activity as compared to primary human hepatocytes. Gene expression of CYP2E1 in UHH over time does not change up to at least culture day 21. This model shows an increased expression of CYP enzymes as compared to the hepatic HepG2 cell line and a more stable expression of CYP enzymes as compared to primary human liver cells.

Taken together, both the ZFE as well as the UHH model are suitable to demonstrate the effect of chemical substances on the KE oxidative stress in the context of this AOP. Although, the appropriateness and the complexity of the UHH model is considered more relevant to humans than the ZFE method since the UHH demonstrates oxidative stress specifically in liver cells.

#### 3.4.1 Organizing the data

The evidence that is gathered for both considerations for each of the elements in the AOP can be briefly described using the developed template and summarized in a table (Supplementary Table S4). Such templates help to identify, collect and organize the data that is required to answer the main question on human relevance.

In addition to the biological evidence, relevant NAMs associated with elements of the AOP under assessment should be described in the template (Supplementary Table S3). Similarly to the biological considerations, we provide a description of each of the considerations that address the relevance of a NAM and provide more context as to what is meant. Evidence obtained from NAMs that are considered relevant for elements of the AOP under study, should be included in the human relevance assessment of the AOP.

We have also created tabulated formats in the template that guide the integration of the evidence within each line of evidence in a categorical manner. Moreover, there are tables provided where the conclusions of each consideration and integrated line of evidence can be collected and organized.

## 4 Discussion

For human health risk assessment of chemicals and pharmaceuticals, information on the relevance of AOPs and associated NAMs to humans is pivotal. To conclude whether or not an AOP is relevant to humans, many different aspects and types of data can (and should) be considered. In order to perform such assessment as transparent and systematically as possible, we modified the human relevance workflow as proposed by Veltman et al. (2025) and provided templates to guide human relevance assessment of an AOP (Figure 1). The current work should be considered as a first step towards a more transparent and harmonized approach for human relevance assessment.

We primarily focused on the question whether an AOP can qualitatively occur in humans. Further questions to assess the human relevance of an AOP regarding quantitative differences remain to be explored. We view this work as a starting point that can and should be adapted in the future. This will, for example, be done in the Partnership on the Assessment of Risks from Chemicals (PARC^4^). Pending questions and challenges encountered during the modification of this workflow are addressed below.

### 4.1 Human relevance workflow – assessment and modifications Q1

In the workflow for human relevance assessment all elements of an AOP should be assessed for each of the proposed considerations. To ensure an efficient search for information, it is recommendable to evaluate a KER with its accompanying KEs. Moreover, in the assessment of certain AOPs it may be appropriate to evaluate combinations of multiple closely related elements, for example, when they belong to the same level of biological organization.

#### 4.1.1 Considerations

The considerations that we propose to assess human relevance are a first step towards a systematic and transparent assessment. These considerations, both biological and empirical, are open for modification, addition or refinement. They may best be evaluated using case studies such as those published for the WHO/IPCS MOA/human relevance framework and additional guidance documents to study biological relevance (e.g., EFSA et al., 2017a). This also applies to the toolbox that was created by Veltman et al. (2025) and supplemented in this work. We envision this toolbox to be an inspirational tool to facilitate the assessment, and not a mandatory or inflexible. Future work may focus on tools to automatically extract relevant information from selected databases or literature. This can increase the objectivity of the assessment.

To evaluate whether empirical evidence from NAMs can be used to assess human relevance of an AOP, the NAM itself should be assessed for its relevance in relation to the element of the AOP that is under assessment. It should be noted that criteria related to the scientific validity of the NAM, such as robustness and reproducibility, are assumed to be sufficient and thus are not included in the considerations. Considering that there currently are no harmonized criteria for the assessment of abovementioned aspects, we considered inclusion of such criteria in the workflow beyond the scope of our work and refer to the assessors’ tool of choice. Before using the evidence obtained from NAMs that are deemed relevant, the quality of the evidence and risk of bias should be assessed, for example, using available critical appraisal tools [e.g., the OHAT Risk of Bias Rating Tool, CATs described by EFSA, the SciRAP tools or ToxRTool (EFSA, 2015; OHAT, 2015; Roth et al., 2021; Schneider et al., 2009)].

It should be noted that the empirical considerations could complement existing templates for technical characterization of NAMs, such as ToxTemp (Krebs et al., 2019) or ReadEDTest (Crouzet et al., 2023), to reduce the amount of duplicate information that is searched for. This will facilitate the human relevance assessment when many of the required information is already reported elsewhere.

#### 4.1.2 Relevance of NAMs

The empirical considerations we suggested will help to identify the strengths and limitations of a NAM with regard to its relevance for an AOP. The resulting information on a NAM is highly valuable with regard to human health risk assessment: it will help to identify NAMs that are well suited for measuring specific KE(s) of a given AOP. Having better insight into the relevance of a NAM (in the context of a specific AOP) will, together with assessment of its validity (OECD, 2005), greatly advance the regulatory use of NAMs for predicting toxicological effects.

#### 4.1.3 Non relevance to humans

In addition to the original workflow, we suggest adding a line of thought to the guidance regarding the evidence for human non-relevance. When information is encountered that supports the AOP not being relevant to humans, this should also specifically be included in the assessment in order to reduce the confidence in the human relevance of an AOP. It should then also be defined when one can conclude that there is a lack of human relevance, i.e., what amount and type of evidence is considered sufficient to not consider an AOP in a human health risk assessment. Thus far not many AOPs are considered not human relevant based on qualitative differences (Meek et al., 2014a). Rather, quantitative species differences will play a role. The magnitude such a difference should have in order for the AOP to be considered not relevant for humans warrants further discussion.

### 4.2 Weight of evidence and integration of evidence

Here we provided a basic suggestion for a weight of evidence approach in the human relevance assessment workflow. For future use, the provided tables with criteria for categorization of the biological and empirical considerations may be refined. These criteria are open for modification and additional considerations or elements may be added or deleted. For each consideration that is added, a line with criteria for categorization should be added as well. Along the same line of thought, criteria for the ‘rule of thumb’ that help to conclude on each line of evidence and the integration of all lines of evidence may be refined.

It may also be considered to extend the three descriptive categories in which the evidence for each of the considerations is categorized. We may follow and adjust the example described by Dekant et al. (2017), where five numerical scores are given to categories, and also includes a null-category that could include evidence for human non-relevance. This weight of evidence approach provides a summarizing score/quality score to which conclusions can be attached based on prior defined cut-off values (e.g., >75% of maximum score results in conclusion x or y, etc.) (Dekant et al., 2017). Similarly, Becker et al. (2017) describe a quantitative weight of evidence approach with positive and negative rating categories. As such, contradictory evidence and lacking evidence can also be included in the human relevance assessment (Becker et al., 2017).

Ideally, a user-friendly tool to evaluate and integrate the evidence may be developed (e.g., along the lines of the SciRAP tool). This tool should also include a visualization of the conclusion on the different lines of evidence and overall integration.

### 4.3 Uncertainty assessment and gap identification

An essential element of a weight of evidence assessment is uncertainty assessment. It is of high importance to understand the uncertainties in the assessment and their possible implications. Furthermore, data gaps in the assessment need to be identified. We have not yet prepared a structured guidance for this. In our view, as a first step the considerations that are required for the human relevance assessment need to be evaluated by the conduct of new case studies. Then, as a next step the uncertainty assessment needs to be included in the guidance according to existing guidance on uncertainty assessment (EFSA et al., 2018; EFSA et al., 2017b; WHO/IPCS, 2018).

### 4.4 Human relevance workflow - Q2 and Q3

To conclude on the human relevance of an AOP for humans, the entire workflow by Veltman et al. (2025) needs to be considered. Here, we provide modifications and suggestions for a weight of evidence assessment concerning the first question in the workflow: can the AOP qualitatively occur in humans? Questions 2 and 3, (“Do human diseases with similar AO have a similar MOA?” and “Is human relevance unlikely in view of quantitative differences?,” respectively) have not been addressed yet. A guided template should also be developed to address the other questions in the workflow. As such, an AOP can be subjected to a complete structured human relevance assessment, from a qualitative and quantitative point of view. Considering that often quantitative differences between species, and not so much qualitative differences lie at the root of human non-relevance (Meek et al., 2014a; WHO/IPCS, 2007), a transparent and structured approach to assessing this is of high importance. However, assessing the quantitative human relevance of an AOP and assessing the quantitative relevance of NAMs is not straightforward to achieve. For example, the magnitude of an effect can differ between genetically diverse cell lines (ICCVAM, 2024). The selection of NAMs in this context is therefore of great importance.

## 5 Conclusion

In this work we present a refinement of the workflow for human relevance assessment of AOPs and associated NAMs. This workflow aids researchers and regulators in the systematic and transparent assessment of human relevance of an AOP. In addition, it helps to evaluate NAMs for their power to provide evidence that supports human relevance assessment. This workflow, along with the developed templates, is a next step towards a harmonized approach of human relevance assessment of an AOP and will greatly facilitate the use of NAMs for human health risk assessment.

## Data Availability

The original contributions presented in the study are included in the article/Supplementary Material, further inquiries can be directed to the corresponding author.

## Footnotes

1
https://www.proteinatlas.org/

2
https://www.ebi.ac.uk/

3
https://www.encodeproject.org/

4
www.eu-parc.eu/
