# Supplementary material for: Refinement of a workflow for human relevance assessment of adverse outcome pathways and associated new approach methodologies
Source: Front Toxicol. 2025 Aug 4;7:1616817. doi: 10.3389/ftox.2025.1616817 (PMC12392633; doi:10.3389/ftox.2025.1616817)
Supplement: Supplementary file 1 [file Supplementaryfile1.docx]

Supplementary Material

**Workflow for Human Relevance Assessment of Adverse Outcome Pathways and Associated New Approach Methodologies**

**Background**

In 2007, Boobis et al. established the mode of action (MOA)/ human relevance framework to assess the relevance of a toxicological pathway to humans (WHO/IPCS, 2007). In 2014, this framework was updated by Meek et al. to extend its utility to emerging areas in toxicity testing and non-testing methods (Meek et al., 2014). In these frameworks, general questions are described that need to be answered to assess the human relevance of a toxicological pathway (e.g. mode of action or adverse outcome pathway (AOP), hereafter referred to as AOP) and the relevance of NAMs related to the key events (KEs) or key event relationships (KERs) of the pathway under study. Yet, a harmonised approach as to how exactly answer these questions is not available. We created a workflow and accompanying guidance and templates with several considerations that can help to answer the questions needed to assess the human relevance of an AOP and the relevance of associated new approach methods (NAMs). The starting point of the workflow is an AOP of which the endpoint is considered relevant for human health risk assessment and for which the overall evidence is considered sufficient (based on the modified Bradford Hill criteria). A toolbox with several types of information and information sources is provided along with a suggestion to perform the weight of evidence analysis to for the human relevance assessment of an AOP.

In general, three questions will need to be answered to assess human relevance (Figure 1). For this workflow, we provide guidance and templates to answer the first question.

**Q1 Is the MIE/KE/KER likely to occur in humans?**

**(Q2 Do diseases with a similar AO have a similar MOA)**

**(Q3** **Is human relevance unlikely in view of quantitative differences?)**


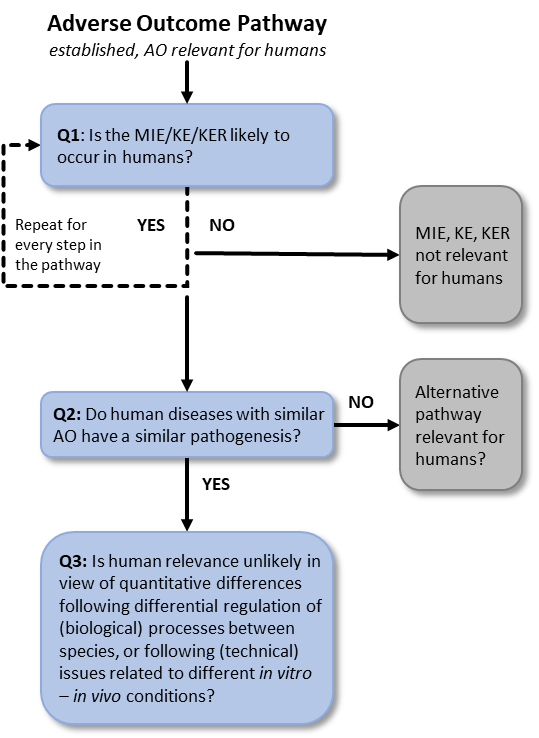


**Figure 1.** Workflow for assessing human relevance of an adverse outcome pathway (AOP) and associated NAMs. AO, adverse outcome; KE, key event; KER, key event relationship; MIE, molecular initiating event. Human relevance assessment systematically evaluates the different elements of an established AOP, of which the AO is considered relevant to humans and of regulatory concern.

**Guidance and templates to the workflow**

**Q1 Is the MIE/KE/KER likely to occur in humans?**

**Assess the qualitative human relevance of the elements in the AOP**

To answer the question if the event is likely to occur in humans, two types of evidence should be pursued; a) information with respect to the underlying biology of the chain of events, and b) the empirical evidence from NAMs that describe the chain of events. Finally, these bodies of evidence should be combined to reach a final conclusion as to the qualitative human relevance of the (elements in an) AOP and the associated test methods.

**PART 1 Biological evidence**

Information on several biological considerations should be used when an element in the AOP is assessed for its human relevance. The identified considerations are 1) target organ, 2) time window, 3) gender/sex 4) clinical data 5) evolutionary conservation.

Shortly describe the event of the AOP under consideration and summarize the supportive data found for the relevance for each biological consideration to humans. The evidence for the relevance of these considerations should be evaluated in as much detail as the available data allow until a clear overall picture of the available evidence is obtained.

**Per individual MIE/KE/KER, various biological considerations can be referred to while assessing the human relevance of the event. Read Table 1 and accompanying guidance and report the relevant information for the considerations for each element of your AOP under assessment by using the guidance below the table.**

| Table 1. Toolbox aiding the human relevance assessment of biological evidence | | |
| --- | --- | --- |
| Considerations | **Tools** | **References** |
| 1. Expression/occurrence in target organ(s) | Expression atlas; ENCODE; human protein atlas; NCBI AceView; NCBI Gene; AOPWiki | https://www.ebi.ac.uk/  https://www.encodeproject.org/  https://www.proteinatlas.org/  https://www.ncbi.nlm.nih.gov/IEB/Research/Acembly/  https://www.ncbi.nlm.nih.gov/gene/  https://aopwiki.org/ |
| 2. Expression/occurrence specific to a sensitive time-window(s); and/or specific to life-stage | Expression atlas; human protein atlas; textbooks; scientific literature; AOPWiki | https://www.ebi.ac.uk/  https://www.proteinatlas.org/  https://aopwiki.org/ |
| 3. Expression/occurrence specific to sex | Expression atlas; textbooks; scientific literature; AOPWiki | https://www.ebi.ac.uk/  https://aopwiki.org/ |
| 4. Relevant case reports, pharmacovigilance (clinical data) | Drug registries (e.g. European Medicines Agency, WHO Vigibase), scientific literature; OMIM; pharmGKB | https://www.vigiaccess.org/  https://www.omim.org/  https://www.pharmgkb.org/ |
| 5. Evolutionary conservation | MSigDB; NCBI HomoloGene | https://www.gsea-msigdb.org/gsea/msigdb  https://www.ncbi.nlm.nih.gov/homologene/ |

**Guidance to the written rationale biological considerations.**

Shortly (in a couple of sentences) describe the element under assessment for its human relevance and the obtained evidence using the biological considerations given below. The sources in the toolbox (table 1) can be accessed in the search for information. Only use the information from individual (epidemiological/ clinical/ experimental) studies for which the relevance and reliability is considered sufficient e.g. based on critical appraisal tools (such as OHAT Risk of Bias, RCT CAT or SR CAT, SciRAP tool, ToxRTool (EFSA, 2015, OHAT, 2015, Roth et al., 2021, Schneider et al., 2009)).

Target organ

This consideration requires information on the actual expression or the occurrence of the element under assessment in the human target organ. Is the evidence supporting the human relevance of the element under assessment? E.g. evidence that the key metabolic process occurs in relevant target organ, and not differences in rate of metabolism.

1. *[Please, shortly describe the evidence for this consideration regarding the element in 3-5 sentences.]*
2. *[Please indicate the tools/links/reference sources that were used (not limited to Table 1)]*

Time-window and life-stage

This consideration requires information on the expression or the occurrence of the element under assessment in a specific time-window and/or life-stage. Is there evidence available to support this element under assessment occurring in humans at the relevant time-window and/or life-stage. E.g. the evidence reflects correct timing in developmental process/key developmental process.

1. *[Please, shortly describe the evidence for this consideration regarding the element in 3-5 sentences.]*
2. *[Please indicate the tools/links/reference sources that were used (not limited to Table 1)]*

Sex

This consideration requires information on the possible (lack of) expression or occurrence of the element under assessment in human males or females. Is the evidence supporting the human relevance of this element under assessment? E.g. sex hormones do (or do not) affect the event under consideration and, therefore, the evidence gathered for the other considerations can(not) be used without constraints.

1. *[Please, shortly describe the evidence for this consideration regarding the element in 3-5 sentences.]*
2. *[Please indicate the tools/links/reference sources that were used (not limited to Table 1)]*

Clinical data

This consideration requires information from case reports, clinical trials or pharmacovigilance. Is there evidence available that supports the element under assessment in relation to the adverse outcome under study in humans? Note that this does not require information on the human occurrence of the AOP as a whole.

1. *[Please, shortly describe the evidence for this consideration regarding the element in 3-5 sentences.]*
2. *[Please indicate the tools/links/reference sources that were used (not limited to Table 1)]*

Evolutionary conservation

This consideration requires information on e.g. the homology of genes or proteins. If the conservation of the element under assessment is very high, expression in humans can be assumed. Describe any information available on the conservation in different species (animal/vertebrate/mammalian).

1. *[Please, shortly describe the evidence for this consideration regarding the element in 3-5 sentences.]*
2. *[Please indicate the tools/links/reference sources that were used (not limited to Table 1)]*

**PART 2 Empirical evidence**

Besides general, ‘textbook’, biology-driven information that can be used to assess human relevance of (elements of) an AOP, also empirical evidence from NAMs can be used as evidence in the human relevance assessment. Each NAM or other model that can be used as source of information, however, should be assessed for its power to support human relevance in relation to measurement of a MIE/KE/KER in the pathway.

Empirical considerations are provided to structure the information in this assessment. Each of the considerations is scored for its supportive power to provide the evidence that can be considered in a human relevance assessment of the respective AOP. The assessment of evidence for all considerations together allow to conclude on the relevance of the specific NAM related to the MIE/KE/KER under investigation and the use of its data for human health risk assessment.

The identified considerations are 1) representativity 2) complexity 3) sensitivity and susceptibility. Shortly describe the method under consideration and the supportive data found.

Next to assessing the NAM’s power to provide evidence for the human relevance assessment, we can, by this assessment, connect NAMs to the pathway under investigation that can be used when assessing the risk of a chemical considering the pathway.

| Table 2. Empirical evidence for human relevance of the MIE/KE/KER. | | | | | |
| --- | --- | --- | --- | --- | --- |
| Stressor | NAM/  Model | Response | Ref. | Considerations | Evidence for human relevance |
| *XX* | *XX* | *XX* | *XX* | 1. Appropriateness:  2. Complexity:  3. Sensitivity/susceptibility : | Weak/ moderate/ strong |
| *Example*  *Ethanol* | *HepG2-E47 cell line* | *Cytotoxicity and oxidative stress in HepG2-E47 cells (CYP2E1 overexpression)* | *Cederbaum 2001* | *1. Human liver hepatocyte cell line, adolescent origin,  2. Immortalized transformed cells*  *3. Many known SNPs, not captured by HepG2 cells (single male donor), not metabolically competent; not captured by HepG2 cells.* | *1. Strong*  *2. Moderate 3. Weak* |
| *^a^* Note that this assessment is not aimed to evaluate the robustness of the NAM or to (pre-)validate the NAM | | | | | |

**Per individual MIE/KE/KER various NAMs or other models can be used to provide evidence for the human relevance assessment of the pathway under study. Please, read Table 2 and accompanying guidance to report the relevant information that a NAM or model can provide for each individual MIE/KE/KER. Indicate the exact response that contributes to the supportive power of the data to the MIE/KE/KER and the references to the studies. Answer the considerations as concise as possible. This table can be copied to cover each element in the AOP.**

**Guidance to written rationale empirical considerations**

Shortly describe the element that is under assessment for its human relevance. Reflect on the NAM/experimental model and its power to provide evidence for the relevance of that element using the considerations below. Usually, specific stressors are used to modulate the element of the AOP and provide the necessary evidence of MIE/KE/KER. The NAM includes both the test system itself and the method of quantification of the endpoint.

*[Please shortly describe here the element of the AOP, the NAM/experimental model under consideration, the stressors used and the exact response related to the MIE/KE/KER. Summarize this in Table 2 and add relevant references]*

1. Appropriateness

Are the experimental model and the measured response appropriate to provide evidence for the relevance of the element of the AOP under study? Consider and describe in relation to element of the AOP and measured response:

- Appropriate organ/tissue type?
- Appropriate life-stage?
- Appropriate sex (optional, if relevant)?
- Appropriate taxonomic class?
- Appropriate response i.e. response directly related to the element of the AOP and reflecting in vivo situation?
- Relevant exposure scenarios possible? E.g. Appropriate transporters included?
- Relevant metabolic activation possible? E.g. similar expression of CYPs compared to WT

*[Please shortly describe the aforementioned aspects of the experimental model and the response regarding this consideration in ~5 sentences.]*

1. Complexity

Is the experimental model complex enough, fit for purpose, to provide evidence for the relevance of the element of the AOP under study? Consider and describe in relation to element of the AOP and measured response:

- Complexity sufficient for level of biological organization? E.g. 2D cell line or whole body
- Physiological state sufficiently captured? E.g. multiple cell types required to measure response?

*[Please shortly describe the aforementioned aspects of the experimental model and the response regarding this consideration in ~5 sentences.]*

1. Sensitivity and susceptibility

Explore what is known on differences in e.g. sex, isoforms, polymorphisms that may affect the element of the AOP under consideration (refer to the Toolbox in pt. 1). Are these aspects covered appropriately in the experimental model and measured response to provide evidence for the relevance of the element of the AOP under study? Consider and describe in relation to element of the AOP and measured response:

- Appropriate expression of element of the AOP? E.g. under or over expression due to cell line/type
- Increased or decreased response due to sex/isoforms/polymorphisms?

*[Please shortly describe the aforementioned aspects of the experimental model and the response regarding this consideration in ~5 sentences.]*

Reliability and validity of the model are considerations that needs to be judged in general, yet does not affect the potential of a NAM to provide evidence that can be considered in a human relevance assessment of an AOP. It should be described what is known on the established use and readiness level of the model. If applicable, add a reference to the test method or protocol.

For each NAM, a profile can be set up that describes its usefulness to provide evidence for human relevance assessment in the context of a specific problem formulation (i.e. element of the AOP under assessment). This can differ depending on the problem formulation.

**Assessing the evidence**

To conclude on the NAM’s supporting power to provide relevant data in relation to measurement of a MIE/KE/KER in the pathway, the NAM needs to be assessed for each empirical consideration. This also provides information on the NAM’s strengths and limitations. The allocation of ‘scores’, however, requires a high level of expert judgement. We suggest a tabulated format where a NAM’s power to support human relevance assessment can be categorised. To objectify the criteria definitions, see Table 3 below.

| **Table 3. Criteria definitions for empirical considerations** | | | |
| --- | --- | --- | --- |
|  | *Weak* | *Moderate* | *Strong* |
| Appropriateness | If model of non-relevant organ/cell type nor life stage concordant to study element in AOP | If model partly relevant to study element in AOP | If model of relevant organ/cell type and life stage concordant to study element in AOP |
| Complexity | If model is not complex enough to study element in AOP | If model is partly complex enough to study element in AOP | If model is complex enough to study element in AOP |
| Sensitivity and susceptibility | If model does not includes relevant differences in sex, isoforms, polymorphisms | If model partly includes relevant differences in sex, isoforms, polymorphisms are the known and correct metabolic system activated | If model includes relevant differences in sex, isoforms, polymorphisms are the known and correct metabolic system activated |

The relevance assessment NAMs depends on expert judgement and whether or not a consideration should carry more (additional) weight. The data from NAMs that are considered relevant can be used as empirical evidence for the human relevance assessment of the MIE/KE/KER. This evidence should support the biological evidence collected in the first part of this workflow.

**PART 3. Integrating and rating the evidence**

To integrate and rate the evidence that is biology-driven and the evidence obtained from NAMs, we suggest a tabulated format to rate the evidence in a categorical manner. Considering that the weighing of evidence requires a high level of expert judgement, criteria/definitions are provided to guide the objectivity in the ranking.

For each consideration, first note if it is applicable to the event under assessment. Then, rate the supportive evidence for human relevance of the event under study using three categories (weak/moderate/strong) using Table 4. The allocation of the scores requires a high level of expert judgement. Consider the direct evidence, indirect evidence and contradictory evidence. To objectify the criteria definitions, see the Table 5 below.

**Fill Table 4 below for each of the considerations, for each of the MIE/KE/KER in your AOP under assessment (copy the table). The human relevance of each MIE/KE/KER is an integration (combination) of the evidence for each consideration, see below.**

| **Table 4. Rating the evidence for human relevance of the MIE/KE/KER/KE** | | | | | | | | |  |
| --- | --- | --- | --- | --- | --- | --- | --- | --- | --- |
| Consideration | Applicable? | | Evidence | | Additional weight? | | Human relevance | |  |
| 1. Expression in organ | | y/n | | w/m/s/- | | +/- | | weak/ moderate/ strong | |
| 2. Expression in dev. stage | | y/n | | w/m/s/- | | +/- | |  |  |
| 3. Differential expression sex | | y/n | | w/m/s/- | | +/- | |  |  |
| 4. Clinical data | | y/n | | w/m/s/- | | +/- | |  |  |
| 5. Conservation | | y/n | | w/m/s/- | | +/- | |  |  |

Use the definitions in Table 5 for each consideration to assess the evidence for human relevance regarding the MIE/KE/KER.

| **Table 5. Criteria definitions for evidence for human relevance** | | | | |
| --- | --- | --- | --- | --- |
| Evidence  Consideration | *Weak* | *Moderate* | *Strong* |  |
| 1. Target organ | Expression of event in target organ cannot be proven | Expression of event in other cells similar to target organ | Expression of event occurs in target organ |  |
| 2. Time window | Expression of event in specific time-window and life-stage cannot be proven | Expression of event in specific time-window and life-stage cannot both be proven | Expression of event occurs in specific time-window and life-stage |  |
| 3. Gender | Expression of event in relevant gender cannot be proven | Contradictory evidence for expression of event in relevant gender | Expression of event occurs in relevant gender |  |
| 4. Clinical data | No clinical data support the event under assessment | Equivocal/contradictory clinical data to support the event under assessment | Clinical data support the event under assessment |  |
| 5. Evolutionary conservation | Event is not highly conserved | Event is conserved only in ‘higher’ species | Event is well-conserved |  |

**References**

EFSA 2015. Tools for critically appraising different study designs, systematic review and literature searches. EFSA Technical Report. DOI: <https://doi.org/10.2903/sp.efsa.2015.EN-836>

Meek, M. E., Boobis, A., Cote, I., Dellarco, V., Fotakis, G., Munn, S., et al. 2014. New developments in the evolution and application of the WHO/IPCS framework on mode of action/species concordance analysis. J Appl Toxicol, 34**,** 1-18. DOI: 10.1002/jat.2949

OHAT 2015. Handbook for Conducting a Literature-Based Health Assessment Using OHAT Approach for SystematicReview and Evidence Integration. Research Triangle PARC, NC: OHAT.

Roth, N., Zilliacus, J. & Beronius, A. 2021. Development of the SciRAP Approach for Evaluating the Reliability and Relevance of in vitro Toxicity Data. Front Toxicol, 3**,** 746430. DOI: 10.3389/ftox.2021.746430

Schneider, K., Schwarz, M., Burkholder, I., Kopp-Schneider, A., Edler, L., Kinsner-Ovaskainen, A., et al. 2009. "ToxRTool", a new tool to assess the reliability of toxicological data. Toxicol Lett, 189**,** 138-44. DOI: 10.1016/j.toxlet.2009.05.013

WHO/IPCS 2007. IPCS Mode of Action Framework Part 1: IPCS Framework for Analysing the Relevance of a Cancer Mode of Action for Humans and Case Studies. Part 2: IPCS Framework for Analysing the Relevance of a Non-Cancer Mode of Action for Humans. World Health Organization.
